# Early adolescent lumbar intervertebral disc injury: a case study

**DOI:** 10.1186/2045-709X-21-13

**Published:** 2013-04-26

**Authors:** Chris T Carter, Lyndon G Amorin-Woods, Arockia Doss

**Affiliations:** 1 School of Health Professions, Murdoch University, Murdoch, Western Australia, Australia; 2 Specialist Radiologist, Image Guided Therapy Clinic, Nedlands, Western Australia, Australia

## Abstract

This article describes and discusses the case of an adolescent male with lumbar intervertebral disc injury characterized by chronic low back pain (LBP) and antalgia. A 13-year-old boy presented for care with a complaint of chronic LBP and subsequent loss of quality of life. The patient was examined and diagnosed by means of history, clinical testing and use of imaging. He had showed failure in natural history and conservative management relief in both symptomatic and functional improvement, due to injury to the intervertebral joints of his lower lumbar spine. Discogenic LBP in the young adolescent population must be considered, particularly in cases involving even trivial minor trauma, and in those in which LBP becomes chronic. More research is needed regarding long-term implications of such disc injuries in young people, and how to best conservatively manage these patients. A discussion of discogenic LBP pertaining to adolescent disc injury is included.

## Background

LBP in children and adolescence is an important and increasing problem, and prevalence increases with age [1]. Systematic review and meta-analysis studies of LBP in adolescence found mean LBP point prevalence and one-year prevalence for adolescents to be around 12%, and 33% respectively [2,3]. Watson et al. [4] reported a one month period prevalence of 24% in schoolchildren aged 11–14 years in northwest England. Historically considered as trivial and non-limiting, LBP in this age-group may have both immediate and long-term consequences for an important proportion of those affected [4]. Risk factors have been debated, although ergonomics of school furniture, school bag weight and mechanics, trauma, history of scoliosis, and involvement of strenuous physical activity may be associative or causative factors in young persons with LBP [5]. There is also increasing evidence that psychological and psychosocial factors may play a significant influence in the aetiology of LBP in this age group [6,7].

Most cases of LBP in the younger population are considered ‘mechanical’ and ‘uncomplicated’, with pathological causes such as neoplasm and infection being very rare [6]. Conservative management and natural history are quite favourable, with the percentage of cases that persist with discomfort longer than one week being less than 15% [5,6]. Therefore, should a young person diagnosed with uncomplicated LBP not respond as quickly as expected to conservative management, pathological causes should again be considered and further clinical examination undertaken. Additionally, less common, non-pathological causes of low back pain such as ‘active’ spondylolysis must be re-introduced into the differential diagnosis [8].

An important and probably underappreciated source of LBP in children and young adolescent persons is the intervertebral disc (IVD) joint. Lumbar disc herniation (LDH), encompassing the categories of protrusion, extrusion, and sequestration is well known to cause LBP [9-11]. Involved in the herniation process are annular tears and other intervertebral joint sources of LBP such as end plate subchondral oedema [9]. For the paediatric patient lumbar disc herniation is less common compared to adults, affecting no more than 5% of the population, and paediatric patients have been shown to constitute only 0.5–6.8% of all patients hospitalized for LDH [12]. Multiple factors have been identified as potential causes, with 30–60% of cases of children and adolescents with symptomatic LDH having had a history of trauma before the onset of their LBP [12].

This case study describes a young adolescent patient with chronic intervertebral discogenic LBP who showed lack of improvement with natural history and conservative management. We discuss intervertebral disc injury in the young population, and clinically relevant pathophysiology of IVD pain.

## Case presentation

### Clinical history and examination

A 13 year old male presented to a private chiropractic clinic complaining of a four month history of localized right LBP with no symptomatic referral into the lower limbs. It was a daily annoyance, particularly worse at night and in the mornings, although not affecting his sleep to any significant degree. His pain was described as “achy and annoying”, not allowing him to sit for long periods due to soreness. He could only remember a trivial fall from his bicycle and landing on his buttocks around the same time his back pain began, but he did not remember it causing him any significant pain or disability at that time. Due to his pain levels he had missed weeks off school, stopped most physical activities, and was taking on average two to four 200 mg non-steroidal anti-inflammatory’s (NSAID’s) daily. He had also been treated sporadically on four occasions at various clinics conservatively with chiropractic care which included high velocity low amplitude (HVLA) and low force spinal manipulation, myofascial release, and exercises. Both NSAID and manual treatment provided only short-term minimal relief. It was obvious that the patient had not likely been following any recommended plan of management from the previous practitioners.

**Figure 1:**
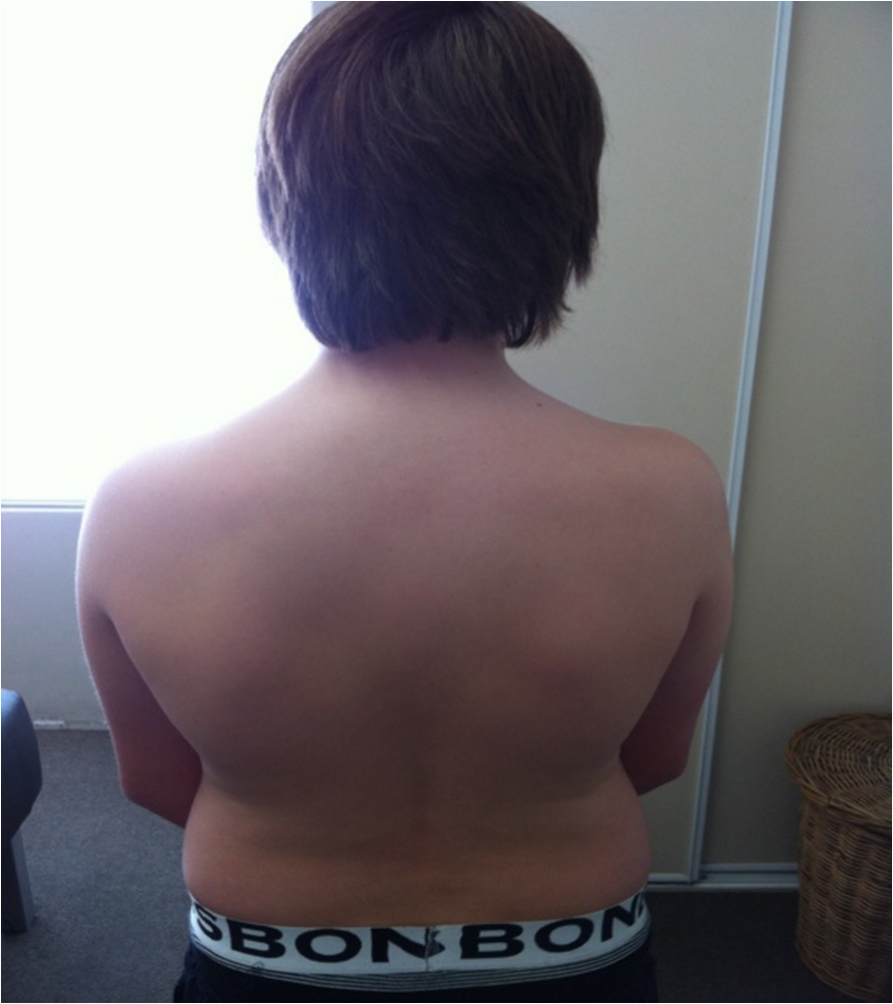
**Posterior view of patient in neutral lumbar ROM.** The posterior view shows a non-distressed young male with no visible scoliosis or antalgia.

**Figure 2:**
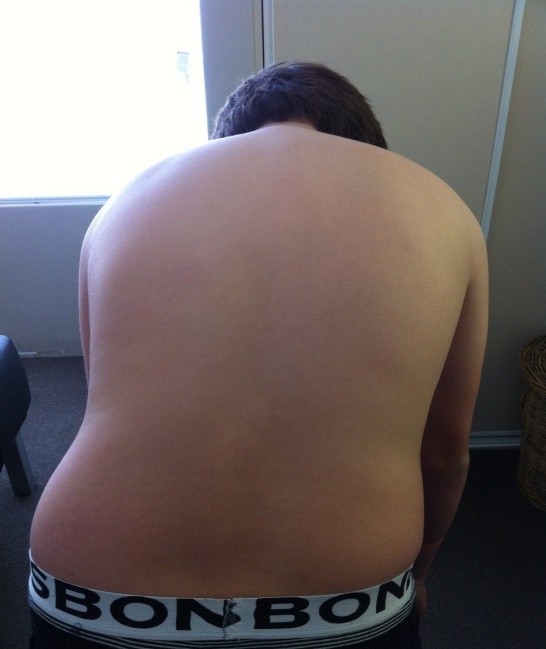
**Posterior view of patient in end-range active flexion lumbar ROM.** This movement reproduced his LBP and created an abnormal right lateral lumbar deviation, appearing to be a possible lumbar scoliotic curve.

Pertinent examination findings revealed a slightly over-weight, non-distressed, adolescent with no significant scoliosis or lumbar antalgia in standing neutral position (Figure 1). When prompted, the patient pointed to the right L4–S1 and sacroiliac joint zones as his main area of discomfort. An Adam’s test was performed to assess for scoliosis, in which the patient was asked to bend straight forward and to try and touch his toes (Figure 2). This movement reproduced his LBP and created an abnormal lateral lumbar deviation to the right, appearing at the time to be a possible lumbar scoliotic curve. The patient also stated he could not flex further than shown due to pain levels and a feeling of significant low back stiffness. Active lumbar range of motion (ROM) was additionally painful in all other ranges, particularly extension, localised at the area of chief complaint. Right lumbar Kemp’s test was positive.

Bilateral lower limb neurological examination was unremarkable, revealing 2+ patellar and achilles reflexes, intact sensation to light touch of L1 to S2 dermatomes, and 5/5 motor testing of L4 to S1 myotomes. Supine examination revealed a Straight Leg Raise (SLR) test that was negative for lower limb radicular pain production, and limited to 45 degrees bilaterally due to significant hamstring tightness. Anatomical leg length measurement (ASIS to medial malleolus) was 90.0 cm right and 91.0 cm left.

Prone examination revealed an estimated right functional short leg of 2.0 cm, and visually his right hemipelvis was significantly cephalad and superior (heightened off the table) oriented, compared to the left hemipelvis. Right sided upper sacroiliac joint compression test was positive for pain and restriction. Left upper sacroiliac joint compression test was negative for pain but was restricted.

Palpation of the right quadratus lumborum, lumbar erector spinae and multifidus muscles revealed significant (3+) hypertonicity and tenderness. Right L4/5 and L5/S1 facet joints were restricted during static and motion palpation. No other orthopaedic and manual physical examination findings regarding the hips, pelvis and lower back were remarkable.

### Diagnosis and radiology findings

A working diagnosis of *‘chronic mechanical low back pain of lower lumbar facet and sacroiliac joint origin’* was made with probable concomitant lumbar idiopathic adolescent scoliosis. Treatment was performed on the first visit consisting of lumbar spinal mobilisations, HVLA manipulation, myofascial release, core stability exercises, and low back education (self-care). He was also referred for lumbar series X-ray due to chronicity of pain levels, assess for scoliosis, and to help rule out pathological causes of his LBP due to the patient reporting consistent night pain. He had not been previously referred for any type of imaging regarding this low back complaint.

**Figure 3:**
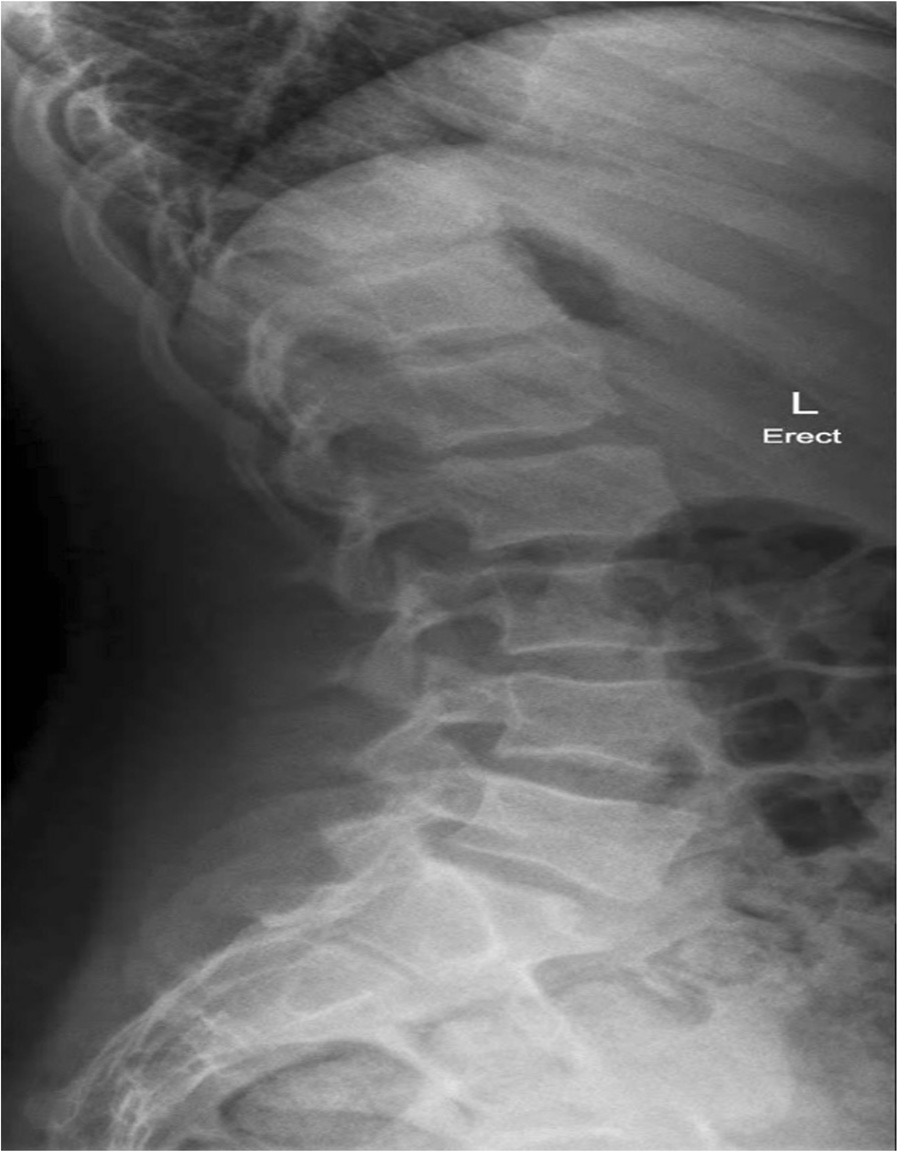
**Lateral lumbar X-ray view.** Note the suspicious area of increased sclerosis in the anterior half of the sacral base.

**Figure 4:**
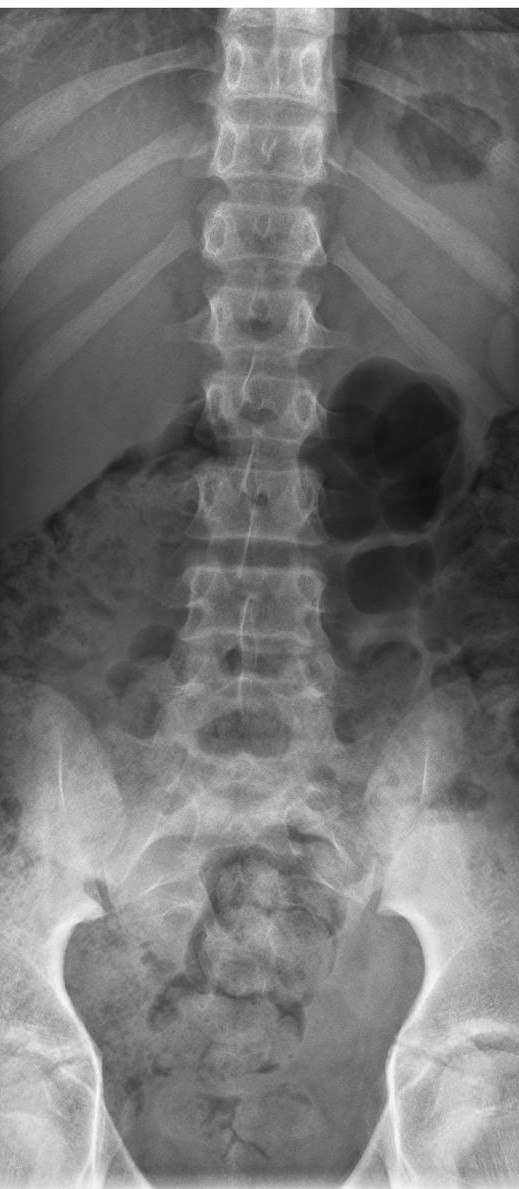
**Antero-posterior X-ray lumbar view.** Note the absence of scoliosis.

The patient represented for the second consultation 11 days later with no change in his LBP or examination findings. Plain film X-ray lumbar spine series (supine) were reviewed and read as ‘normal’ findings by the local hospital imaging centre radiologist. Further chiropractic review of the lateral lumbar view revealed a remarkable small increase in bony sclerosis in the anterior half of the sacral base (Figure 3). Of further interest, was the absence of scoliotic curvature on the AP lumbar view (Figure 4). Considering his abnormal functional right sided antalgia during flexion ROM and no X-ray evidence of scoliosis, the possibility of an IVD injury was considered more likely. This was particularly appropriate given the history of initial injury being a compressive axial force through the lumbar spine after falling off his bicycle, and chronicity of pain. It was postulated there could have been enough force to cause injury to the lumbar IVD’s, vertebral body endplate’s, or even the vertebral body.

The patient was subsequently referred for MRI examination of the lumbar spine by the chiropractor to a private facility on the second visit to assess for lower lumbar annulus fibrosus lesion, disc herniation and inflammatory adjacent marrow oedema. These lesions were considered possible pain-inducing candidates given the patients clinical picture of chronicity, painful antalgia and night pain.

**Figure 5:**
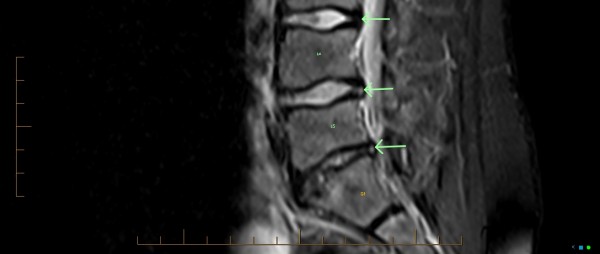
**MRI, sagittal STIR midline image.** Annular tears are seen as posterior annulus fibrosus hyperintensity (bottom two arrows pointing to white dots) at L4/5 and L5/S1 discs. Compare with low signal (dark) of the normal L3/4 disc (top arrow).

**Figure 6:**
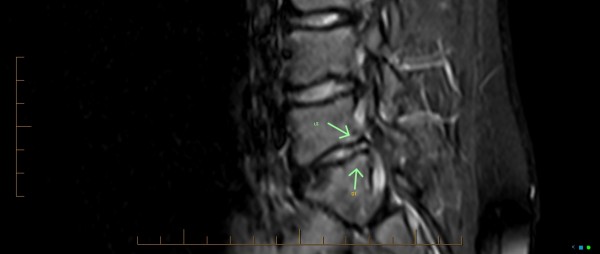
**MRI, sagittal STIR view.** The L5/S1 subchondral bone marrow hyperintensity (areas of white) seen on both sides of the L5/S1 disc (arrows), is suggestive of annular injury and ongoing inflammatory response. L4/5 right paramedian disc protrusion, and L5/S1 disc height reduction with dehydration and right posterolateral disc protrusion that appears chronic. Anterior sacral base bone marrow hypointensity indicates chronic marrow repair.

In this case, lumbar MRI evaluated by the private facility medical radiologist, revealed several clinically relevant findings as a probable cause of his resilient LBP. Figures 5 and 6 show several intervertebral disc joint lesions that are of plausible clinical significance. Figure 5 shows two annular tears that can be seen posteriorly as annulus fibrosis hyperintensity, known also as high intensity zone (HIZ), at the L4/5 and L5/S1 discs. The L4/L5 and L5/S1 discs also reveal a shallow focal disc protrusion. At L5/S1, disc height reduction, loss of signal intensity, and mild adjacent L5 and S1 subchondral marrow oedema is seen. Figure 6, reveals a sizeable area of hypointensity (from past intraosseous herniation) seen in the anterior half of the sacral base. This represents chronic marrow repair, most probable from past compressive trauma to the area. Interestingly, this area of damage correlates to the line of sclerosis seen on the AP plain film X-ray.

### Case management

Twenty-one days post initial consultation, the patients third consultation consisted of a review of the MRI findings, as well as a recommendation to the mother that her son attends the additional opinion of a paediatric spine specialist for co-management. The short and long term prognosis of this patient’s low back condition was of utmost importance, particularly as the patient was already in initial stages of puberty and therefore his growth will be highly accelerated in the following years. His antalgia and chronic low back pain were significantly affecting his quality of life, and his chronic use of NSAID’s in itself constituted a health risk [13]. Referral to a specialist for co-management was deemed the most appropriate short-term measure, in addition to conservative management which would focus primarily on tissue-sparring injury education, postural pain management, core and hip stability rehabilitation, flexibility, and chiropractic passive care. The patient was referred to his medical General Practitioner via letter with a detailed description of clinical situation and imaging results, recommending subsequent specialist referral. Following the third consultation, the patient did not return for further chiropractic management.

Further verbal follow-up with the patient and his mother two months later revealed that the patient was to see a specialist shortly. Five months later, the lead author received a clinical update letter from a specialist paediatric rheumatologist dated 10 months post LBP commencement. This letter indicated that the patient had been sent for blood tests which revealed a completely normal full blood count, ESR, ANA and Rheumatoid Factor. He had been diagnosed with ‘Lumbar Spondylitis’, was no longer taking Naproxen, and had been sent for a hospital based physiotherapy rehabilitation course of management involving stretches and strengthening exercises. The patient reported that he was significantly better over the last several months, with only occasional niggles, but still had LBP and limited movement down his right leg with ROM. Paediatric rheumatologist examination revealed that he still had a rather stiff lumbar spine with limited forward flexion and slight discomfort with extension but good thoracic rotation. There was no tenderness and his peripheral joints were completely normal. Assessment summary revealed that currently things were stable, but that he was still somewhat limited. A repeat MRI was to be completed shortly with a subsequent follow up examination. This patient’s second lumbar MRI, 10 months post LBP commencement, revealed ‘unaltered changes at L4/5 and L5/S1 since the initial MRI’. The lead author only had access to the report, and there was no mention on this report regarding subchondral oedema or annular tears (HIZ’s).

No further information regarding this patient was received. It can therefore be assumed that although his LBP and ROM improved significantly after ten months, he still had LBP and sub-optimal low back function. To any health practitioner, this would be considered not normal for a 14 year-old boy, and worrisome for the long-term functional health of his lumbar spine.

## Discussion

Most childhood and young adolescent LBP cases are uncomplicated in origin and mechanical in nature [6]. Patients tend to respond quickly and successfully to natural history and/or conservative management, and their back pain only uncommonly progresses to become chronic. Childhood development of chronic LBP is one of the major reasons for chronic LBP in adulthood [7]. Children experiencing persistent or recurrent chronic pain have been shown to miss school, and have higher chance of developing conditions such as depression and anxiety [1].

This case study highlights the importance of recognizing the intervertebral disc, vertebral end plates, and subchondral vertebral bone as possible sources of LBP in the young patient. If LBP becomes chronic and unresponsive to conservative management, these structures must be considered as possible sites of nociceptive origin and resultant disability. This assumption is not meant to minimalize or forget the effects of neurological peripheral and/or central sensitizations role in chronic LBP, which is beyond the discussion this case study’s clinical importance it is trying to achieve.

The chronicity of this patient’s pain and mild relief from conservative management were indications that his LBP was more complex in origin, compared to typical mechanical LBP. The antalgia seen during physical examination motion evaluation was abnormal for a 13 year old, and could not be attributed to a scoliosis. It is unusual for antalgia to be caused by neural or other pernicious pathology [14]. Therefore, it is prudent to consider an intervertebral disc injury as a cause of abnormal painful antalgia in the young adolescent population. Zhu et al. [15] reviewed the clinical features and treatment strategy of LDH in adolescents initially misdiagnosed as idiopathic scoliosis. All patients had initially presented with a scoliotic curve as their chief complaint, yet curvature and disability did not resolve until surgical management of the affected discs was undergone.

Many paediatric and early adolescent lumbar disc injuries have been historically attributed to trauma such as falls and sports-related trauma [12]. However, it is becoming more widely regarded that, as in adult LDH, in some cases minor trauma and/or repetitive biomechanical stress may be the provocative event in the exacerbation of a disc that is already herniating and/or undergoing degeneration due to genetic predisposition [11,12,16,17]. Interestingly, up to 57% of adolescent LDH patients have a first degree relative with a lumbar herniation history [11,12].

Zhu et al. [15] found a high incidence of significant hamstring tightness in adolescent LDH patients, although, it is still unclear whether hamstring tightness is an inducing or ensuing factor of LDH. Our patient had very prominent posterior lower limb myofascial tightness, limited to 45 degrees during SLR test bilaterally.

In this case, MRI was helpful in showing annulus fibrosus lesion via HIZ’s, LDH, and inflammatory adjacent marrow oedema. It is reasonable to postulate that these lesions seen on his MRI were significantly involved causes for his chronic LBP. It has been proven that some disc lesions in adults, even when visualized on MRI, are not clinically symptomatic [18]. Kjaer et al. [19] undertook a large study of 439 thirteen-year-old children and assessed lumbar MRI findings. One third of the children showed some sort of lumbar degenerative disc sign, most commonly loss of disc signal intensity. Interestingly, only 24 (5%) had HIZ’s, 11 (3%) had a disc protrusion, 1 (0.5%) had a type 1 Modic change, and 0 (0%) had a type 2 Modic change. Positive associations between degenerative disc findings and self-reported LBP were found, and there was a strong association between those children that ‘sought care of a practitioner for their LBP’ and disc protrusions and HIZ’s. In our case, considering the mechanism of original injury to the lumbar spine some four months prior, and lack of improvement with treatment and natural history, it was assumed that there was reasonable causal relationship between the mechanism of the fall, the intervertebral disc and endplate injuries found on MR Imaging, and his chronic LBP.

Most anatomical elements of the lumbar spine and its surrounding soft tissue network contain nociceptive innervations that may be origins of LBP. It is widely accepted that both the outer annulus fibrosus of the IVD and cartilaginous vertebral endplate are sources of discogenic LBP [17]. Discogenic pain can arise from structural failure and bulging of the disc [17]. Our patient’s most uncomfortable daytime position for LBP was sitting. The lumbar disc space pressure is highest in the sitting position, thus putting increased physical and gravitational stress on the patient’s annular tissue and endplates, thus causing mechanical stretch of nociceptors in these areas [20].

Discogenic LBP also originates from nociceptive neural and vascular in-growth in the outer annulus, into annular tears, and can cause peripheral sensitisation [17]. These inflammatory factors may have likely been of partial cause of our patients’ night and morning pain and stiffness.

Degenerative vertebral endplate injury and subchondral bone marrow oedema seen on MRI are known as Modic changes, and are arguably a source of LBP [21,22]. Figure 6 showed the subchondral bone marrow hyperintensity on both sides of the posterior half of the L5/S1 disc which is reflective of inflammatory bone marrow annular injury and ongoing inflammatory response in the subchondral bone (Type 1 Modic). It also showed subchondral marrow hypointensity seen in the anterior half of the sacral base, which is reflective of marrow histological change (Type 2 Modic). Currently, only type 1 changes have been proven to be a source of LBP [22]. It is possible that at least some of this patient’s LBP can be attributed to the changes associated with the modic changes occurring at his vertebral endplates, and therefore his pain could be of vertebral endplate and subchondral bone origin. As stated previously, our patient had a sizeable area of Type 2 modic change in the sacral base, and this was not found in any of 439 thirteen year-old children in the Kjaer et al. [19] study.

A review by Dang et al. [12] of current treatment for lumbar disc herniation made recommendations regarding management of childhood disc injury. They stated that conservative management of adolescent disc injury is the first choice treatment, particularly in those patients without significant neurological deficits. A growing spine is at risk to surgical trauma and other iatrogenic reactions that can develop after surgical intervention. However, with regards to LDH, current evidence agreed that success with conservative management for adolescent LDH is not as effective as it is for adults, and surgical complications are relatively rare.

Currently, there is a lack of evidence regarding best practice conservative management for adolescent intervertebral disc injury when there is no significant neurological deficit. This is most likely due to the fact that this is not a common clinical condition in everyday practice. There are numerous manual therapy techniques that may be well suited for a trial of conservative management for this type of back pain presentation. The discussion of these types of therapies, as well as other non-surgical therapies for discogenic LBP, such as percutaneous injections, is well beyond the means of this article.

In our case, it was seen prudent for referral to a paediatric specialist, due to the fact that the patient had not followed any recommended course of conservative management, lack of natural history improvement, injuries sustained to the lower lumbar spine, and development of chronic LBP. This referral helped ensure all appropriate clinical measures were taken to limit long term IVD injury consequences in his rapidly growing spine and help prevent internalizing behaviour change. This case highlights the importance of a multidisciplinary management of a young person suffering from chronic discogenic LBP.

## Conclusion

The prevalence of childhood and adolescent LBP is more common than once thought. Discogenic pain from annular tears, herniation and vertebral endplate injury, must be considered particularly in cases involving chronic LBP, and lack of response to conservative manual therapy. MRI can be useful in appropriately connecting patient history, physical examination, and imaging findings to correlate the most likely cause of a patient’s LBP. It should be used particularly when conservative management and/or natural history fails to resolve the LBP disorder. Specialist referral is warranted when pain levels are intractable or when continuous pain and disability occurs after a course of conservative management. Speedy resolution of lumbopelvic function is considered important to help limit any long term deleterious effects on spinal growth and therefore improve quality of life in young persons. More research is needed regarding long-term implications of IVD injuries in people of such young age, and how best to conservatively manage them.

## Consent

Written informed consent was obtained from the patient’s legal guardian [mother] for publication of this case report and any accompanying images. A copy of the written consent is available for review by the Editor-in-Chief of this journal.

## Competing interests

The authors declare that they have no competing interests.

## Authors’ contributions

CC cared for the patient, performed the literature review, and prepared the manuscript. LA-W performed the literature review and assisted in the preparation of the manuscript. AD assisted in radiological interpretation, and authored the MRI results section. All author’s read and approved the final manuscript.
